# Emerging adults’ neuroticism links with depressive symptoms through personal belief in a just world and forgiveness

**DOI:** 10.3389/fpsyg.2025.1489371

**Published:** 2025-06-18

**Authors:** Sixiang Quan, Huibin Kang, Xiaohui Yang

**Affiliations:** ^1^Beijing Key Laboratory of Learning and Cognition, Research Center for Child Development, School of Psychology, Capital Normal University, Beijing, China; ^2^College of Education, Capital Normal University, Beijing, China; ^3^Shaanxi Provincial Key Research Center of Child Mental and Behavioral Health, School of Psychology, Shaanxi Normal University, Xi’an, China

**Keywords:** neuroticism, personal belief in a just world, forgiveness, depressive symptoms, emerging adults

## Abstract

High neuroticism is a stable risk predictor of depressive symptoms. Researchers struggled to understand how neuroticism linked with depressive symptoms. The aim of the current study was to explore whether personal belief in a just world and forgiveness acted as mediators in the relations between neuroticism and depressive symptoms among emerging adults. A total of 630 non-clinical undergraduates completed questionnaires about the studied variables. A sequential mediation model with personal belief in a just world and forgiveness as mediators of the association between neuroticism and depressive symptoms was explored. The results found both direct and indirect effects of neuroticism on depressive symptoms. The sequential mediation analyses revealed that the positive relationship between neuroticism and depressive symptoms was simply and sequentially mediated by personal belief in a just world and forgiveness. These results suggested that neuroticism increased the risk of depressive symptoms by reducing the personal belief in a just world and forgiveness, as well as by the sequential mediating effects of personal belief in a just world and forgiveness. The implementing interventions specifically focused on elevating the level of personal belief in a just world and forgiveness may be important for ameliorating the depressive symptoms.

## 1 Introduction

Depression is a prevalent psychiatric disorder worldwide. According to the World Health Organization, depression affects more than 264 million individuals worldwide, is the leading cause of disability and is a significant contributor to the overall global burden of disease (WHO, 2020). In China, approximately 54 million individuals suffer from depression, and the lifetime prevalence of depression is 6.8% (Huang et al., 2019). Depressive symptoms are characterized by low or negative mood, diminished interest in previously enjoyable activities (Rutter et al., 2013). Emerging adulthood, defined herein as age 18 to the late 20s, is an important time for self-discovery, as many individuals will live independently for the first time, attend college, and engage in formal romantic relationships during this developmental stage (Arnett, 2014). Although young adulthood has traditionally been viewed as a respite from earlier adolescent “storm and stress” (Galambos et al., 2006), many patterns of depressive symptoms persist or emerge during this stage (Reed-Fitzke, 2019). Depression is associated with psychiatric comorbidities (Ayuso-Mateos et al., 2010) and significant psychosocial impairment, including decreased well-being (Malone and Wachholtz, 2018), decreased quality of life (Gao et al., 2017), and poor relationship quality (Vujeva and Furman, 2011), in emerging adulthood. Therefore, it is importance to examine how preexisting risk factors such as personality traits forecast depression during this developmental stage. Tracing the pathways between personality traits and depression can help elucidate proximal processes involved in the development of mood disorders.

### 1.1 Neuroticism and depressive symptoms

Personality traits were found to play important roles in depressive symptom onset (Gong et al., 2020). Specifically, neuroticism was identified as one of the strongest predictors of depressive symptoms (Hakulinen et al., 2015). This personality trait was characterized by the tendency to experience negative affect over time, especially when a person was threatened, frustrated or facing loss (Barnhofer and Chittka, 2010). Many studies found the positive link between neuroticism and depression (Kotov et al., 2010; Ormel et al., 2013; Speed et al., 2019). Moreover, a twin study (Kendler et al., 2006) and genome-wide association study (GWAS) (Chu et al., 2020) revealed that neuroticism was genetically related to depression.

Although the positive relation between neuroticism and depressive symptoms was well established, the underlying mechanism between these two variables remained further examined. The predisposition model of the relation between personality and mood disorders demonstrated that individuals with high neuroticism were at increased risk for subsequently depression and that other variables played a role in mediating or moderating this transition (Klein et al., 2011). According to the biopsychosocial model of health psychology (Sarafino, 2008), coping strategies mediated the relation between dispositional characteristics, such as neuroticism, and health-related quality outcomes. Previous empirical studies confirmed the mediation role of coping strategies between neuroticism and depressive symptoms (Chen et al., 2020; Muris et al., 2005; Roelofs et al., 2008; Yoon et al., 2013). However, these studies predominantly focused on the mediation effects of maladaptive coping strategies such as rumination (Chen et al., 2020; Muris et al., 2005; Roelofs et al., 2008) and worry (Muris et al., 2005). The greater tendency to adopt maladaptive coping strategies does not imply the absence of adaptive coping strategies. Although some studies focused on the mediation effects of adaptive coping strategies between these two variables, most of them focused on individuals’ general affect coping ability (Andrés et al., 2016; Su et al., 2018; Yoon et al., 2013). Few studies focused on the specific adaptive coping strategies as the mediator between neuroticism and depressive symptoms among emerging adults. Targeting specific adaptive coping strategies was helpful to development of more fine-tuned intervention programs. This study aimed to investigate the role of two specific adaptive coping strategies including personal belief in a just world (PBJW) and forgiveness and examined the mediation role of these two variables between neuroticism and depressive symptoms among emerging adults.

### 1.2 Personal belief in a just world as a mediator

Personal belief in a just world, defined as individuals’ belief that the events in life are just and orderly (Lerner, 1980), is considered a positive and healthy coping mechanism (Furnham, 2003). Just world theory advocated that people believed that they get what they deserve and deserve what they get (Lerner, 1980). In other words, it suggested that being a good person led to having good things, and that being a bad person led to having bad things (Furnham, 2003). However, a meta-analysis revealed that neuroticism was negatively associated with PBJW (Nudelman, 2013). Those with high scores in neuroticism were more likely to experience anxiety, and anxiety may hinder these individuals from developing a notion of a stable, orderly environment, which formed the bias for a just world perspective (Lerner and Miller, 1978; Nudelman, 2013) and then inhibited the development of PBJW.

As a powerful personal coping resource, PBJW has important adaptive functions that help individuals cope with critical life events. The stronger PBJW an individual possesses, the better he or she can be expected to cope (Xie et al., 2011). PBJW provides a framework for interpreting the events in one’s life and consequently has effects on mental health (Dalbert, 1999, 2001). A deficit in PBJW makes individuals evaluate events in their life as more unfair and results in a worse state of mental health, such as more depressive symptoms and lower psychological well-being (Valiente et al., 2010). Stronger PBJW allows individuals to maintain a stronger sense of mastery or control of the world and to evaluate negative events as less unfair, thus resulting in fewer depressive symptoms (Dalbert, 2001; Fischer and Holz, 2010; Valiente et al., 2010). PBJW may be an intervening variable and mechanism that could prevent or delay depressive symptoms (Carifio and Nasser, 2012). Therefore, PBJW is a likely candidate for mediating the relation between neuroticism and depressive symptoms. However, no study to date directly explored this relation.

### 1.3 Forgiveness as a mediator

Forgiveness refers to individuals’ deliberate attempts to reduce unhappy feelings and thoughts to improve their happiness, which usually requires them to reframe negative events or experiences as positive or neutral (Thompson et al., 2005). According to the transactional model of stress and coping (Lazarus and Folkman, 1984), individuals appraise identical events differently and their subjective appraisals are determinants of mental health outcomes. Forgiveness, as an adaptive emotion-focused coping strategy, facilitates cognitive and emotional reframing of negative experiences and then promote individuals’ mental health. Thus, forgiveness may mediate the relation between neuroticism and depressive symptoms.

Neuroticism has been proposed to be the higher-order personality construct that have most consistently contributed unique variance to forgiveness-related variables (McCullough and Hoyt, 2002; Walker, 2017). As neuroticism predisposes individuals to perceive events negatively, neurotic individuals are easily offended and angered. Consequently, they tend to experience negative affect more frequently and exhibit greater emotional fluctuation (McCullough and Hoyt, 2002). Forgiveness mitigate health risks and enhance health resilience (Worthington and Scherer, 2004). Empirical studies suggested that forgiveness negatively predicted depressive symptoms and played a key role in reducing depressive symptoms (Burnette et al., 2009; Chung, 2016). Therefore, based on these theoretical and empirical studies, it is reasonable to speculate that forgiveness mediates the relationship between neuroticism and depressive symptoms.

### 1.4 The sequential mediation effects of personal belief in a just world and forgiveness

Individuals who focus on personal justice are more likely to have a forgiveness tendency (Bartholomaeus and Strelan, 2016; Strelan, 2007). PBJW is correlated with various adaptive and approach-oriented outcomes (Bartholomaeus and Strelan, 2019), the most notable of which is forgiveness (Strelan and Sutton, 2011). Justice motive theory asserts that concern for justice is based on a “personal contract” between individuals and the environment. In the development of their personal contracts, individuals become convinced that unfairness or transgression that they suffer is reasonable (Lerner, 1997). In line with this belief, individuals with strong PBJW seek to behave in accordance with their belief (Hafer, 2000). They believe that the world treats them fairly and decently; thus, they are more likely to use forgiveness to restore their PBJW when they encounter unfairness or transgression (Bartholomaeus and Strelan, 2016). Moreover, McCullough et al. (1998) suggested that personality traits are a distal predictor and that feelings about transgressors are a proximal predictor of forgiveness. Therefore, whether neuroticism is linked with depressive symptoms through the sequential mediation effects of PBJW and forgiveness was examined in the current study.

### 1.5 The present study

In summary, although the direct relation between neuroticism and depressive symptoms was well established, the underlying psychological mechanism required further investigation. Revealing the mechanism between these two variables could improve our understanding of why and how neuroticism is linked with depressive symptoms. Thus, the aim of this study was to examine a sequential mediation model among Chinese emerging adults. In particular, we proposed the following three hypotheses.

**Hypothesis 1:** PBJW simply mediates the relation between neuroticism and depressive symptoms.

**Hypothesis 2:** Forgiveness simply mediates the relation between neuroticism and depressive symptoms.

**Hypothesis 3:** PBJW and forgiveness sequentially mediate the relation between neuroticism and depressive symptoms.

## 2 Methods

### 2.1 Participants

This study recruited 630 undergraduates (304 females and 326 males, M_*age*_ = 19.82, SD = 1.38) from three universities in northern China via an advertisement. All participants completed the questionnaires online and signed an informed consent form before their participation. Each participant received a small gift when they finished the questionnaires.

### 2.2 Measures

#### 2.2.1 Neuroticism

We adopted the Chinese version of the Neuroticism Subscale of the NEO Five-Factor Inventory (Leung et al., 1997), which was developed by Costa and McCrae (1992) to assess the level of individuals’ neuroticism. It contains 12 items, and each item is rated on a 5-point scale ranging from 1 (strongly disagree) to 5 (strongly agree). The average scores of these items were calculated. A higher score represents a higher level of neuroticism. The Cronbach’s α was 0.82 in the current study.

#### 2.2.2 Personal belief in a just world

We adopted the Chinese version of the Personal Belief in a Just World Scale (Su et al., 2012), which was originally developed by Dalbert (1999) to assess the personal belief that events in one’s daily life are just. It contains 7 items, and each item is rated on a 6-point scale ranging from 1 (strongly disagree) to 6 (strongly agree). The average scores of these items were calculated. A higher score represents stronger PBJW. The Cronbach’s α was 0.92 in the current study.

#### 2.2.3 Forgiveness

We adopted the Chinese version of the Heartland Forgiveness Scale revised by Wang (2006), which was originally developed by Thompson et al. (2005) to assess a dispositional tendency toward forgiveness. The Chinese version of this scale consists of 24 items categorized into two dimensions: forgiveness of self (12 items) and forgiveness of others (12 items). Each item is rated on a 7-point scale ranging from 1 (almost always false for me) to 7 (almost always true for me). Total scores of the items were calculated. A higher score represents a higher dispositional tendency toward forgiveness. The Cronbach’s α was 0.81 in the current study. Following prior studies (e.g., Arslan, 2017; Lucas et al., 2010; Xia et al., 2017), forgiveness was modeled as a latent variable with the forgiveness-self and forgiveness-others as observed indicators to reduced the potential influence of measurement errors.

#### 2.2.4 Depressive symptoms

We adopted the Chinese version of the Center for Epidemiologic Studies Depression Scale, which was designed by Radloff (1977) specifically for use with non-clinical samples to assess the severity of participants’ depressive symptoms in the previous week. The scale contains 20 items, and each item is rated on a 4-point scale ranging from 0 (rarely or less than 1 day) to 3 (most of the time or more than 5 days). The responses were summed to calculated the total score. The cut-off score is set at 16 to identify the clinical level of depression. However, researchers cautioned using this cut-off score, as it was associated with a high false positive rate in non-clinical samples (e.g., Yoon et al., 2013). Thus, in this study each participant was included and a higher score represented more depressive symptoms during the previous week. The Cronbach’s α was 0.92 in the current study.

### 2.3 Data analysis

The data were analyzed in two steps. First, preliminary analyses of common method variance and descriptive and correlation analyses were conducted in SPSS 22.0 and Mplus 8.1 (Muthén et al., 1998–2017). Second, the sequential mediation model was examined in Mplus 8.1. Neuroticism, PBJW, and depressive symptoms were treated as manifest variables while forgiveness was treated as a latent variable with the scores of two subscales as observed indicators in the structural equation models. In addition, we adopted the bootstrapping method, which does not consider the data distribution, to estimate the confidence intervals (CIs) of the indirect effect, and 5000 bootstrap samples were generated. In all analyses, age and gender were controlled as covariates because they have been found to be correlated with depression (Frey et al., 2020; Gao et al., 2019).

## 3 Results

### 3.1 Preliminary analyses

#### 3.1.1 Common method variance test

Harman’s single-factor test was conducted to assess common method bias (Podsakoff et al., 2003). We obtained 11 factors with eigenvalues greater than 1, determined by exploratory factor analysis with unrotated factor solution. The first factor explained 21.44% of the variance. Furthermore, confirmatory factor analysis showed that the 1-factor model had poor fit: χ^2^/df = 36.05, CFI = 0.67, TLI = 0.57, RMSEA = 0.24. These results suggested that common method variance was not a serious problem in the current study.

#### 3.1.2 Descriptive statistics and correlation analyses

The means, standard deviations, and correlation coefficients of the variables are presented in Table 1. The results show that neuroticism was significantly positively correlated with depressive symptoms and significantly negatively correlated with PBJW and forgiveness. PBJW was significantly positively correlated with forgiveness and significantly negatively correlated with depressive symptoms. Forgiveness was significantly negatively correlated with depressive symptoms.

**TABLE 1:** Descriptive statistics and correlations between variables.

Variables	*M*	SD	1	2	3	4
1 Age	19.82	1.38	1			
2 Neuroticism	2.90	0.59	−0.12**	1		
3 PBJW	4.34	0.78	0.03	−0.31***	1	
4 Forgiveness	100.37	12.43	0.03	−0.42***	0.34***	1
5 Depressive symptoms	18.72	8.88	−0.02	0.59***	−0.40***	−0.45***

*N* = 630. **p < 0.01, ***p < 0.001.

### 3.2 Sequential mediation analyses

**FIGURE 1:**
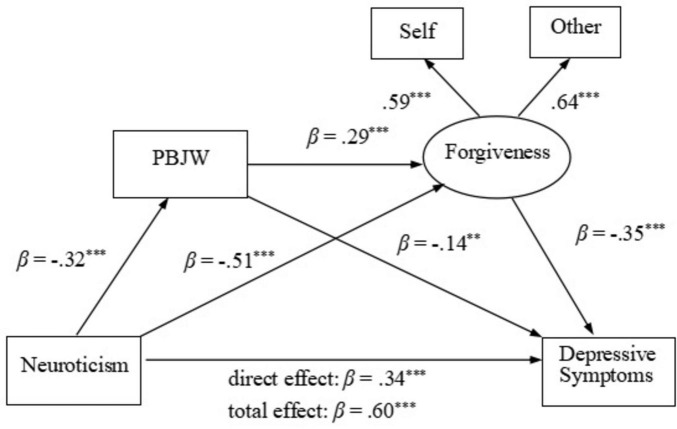
Path coefficients for effects of neuroticism on depressive symptoms. *N* = 630. All the path coefficients are standardized. **p < 0.01, ***p < 0.001.

We first examined the relationship between neuroticism and depressive symptoms and the possible mediating roles of PBJW and forgiveness. The hypothesized model with all direct paths between the study variables showed an accepted fit to the data. The indices were as follows: χ^2^/*df* = 2.02, CFI = 0.99, TLI = 0.97, RMSEA = 0.04, SRMR = 0.02. All direct path coefficients among the variables were significant. The path coefficients are presented in Figure 1.

Then, we used a bootstrap estimation procedure to test the significance of the mediating effects. The indirect effects and their associated 95% CIs are presented in Table 2. Significance was determined if the 95% CI did not include zero. The bootstrapping results suggested that all indirect effects were significant. Specifically, neuroticism indirectly affected depressive symptoms via PBJW (neuroticism → PBJW→ depressive symptoms) and forgiveness (neuroticism →forgiveness →depressive symptoms) separately and sequentially (neuroticism →PBJW → forgiveness→ depressive symptoms). Therefore, hypotheses 1–3 were supported.

**TABLE 2:** Bootstrap test for indirect effects from neuroticism on depressive symptoms.

Model pathways	Estimated	95%CI	
		Lower	Upper
Neuroticism→ PBJW→ depressive symptoms	0.05	0.01	0.08
Neuroticism→ forgiveness→ depressive symptoms	0.18	0.10	0.33
Neuroticism→ PBJW→ forgiveness→depressive symptoms	0.03	0.02	0.06

*N* = 630. CI, confidence interval.

## 4 Discussion

Emerging adulthood is an special period characterized by both opportunity and vulnerability. Individuals during this stage experienced multifaceted stressors (Arnett, 2014). These difficulties may be internalized as depressive symptoms. A large-scale survey indicated that the prevalence of depressive symptoms peaks during emerging adulthood (Sun et al., 2025). Given that this period sets the stage for later adult adjustment and functioning, identifying the possible onset mechanism of depressive symptoms during this period was particular importance. Neuroticism is a well-established predictor of depressive symptoms. According to biopsychosocial model, coping strategies play a mediation role between neuroticism and depressive symptom. Individuals’ coping ability varied across time, as it taken a certain level of cognitive and emotional maturity to assess the situations or events they encountered (Carbonell et al., 2005). Thus, examined the mediation effects of PBJW and forgiveness between neuroticism and depressive symptoms among emerging adults were especially meaningful. The results confirmed that neuroticism was positively related to emerging adults’ depressive symptoms through the simple mediation effect of the adaptive coping resources PBJW and forgiveness and the sequential mediation effect of these two variables. Specifically, neuroticism may weaken emerging adults’ PBJW and forgiveness, and decreased PBJW and failure in forgiveness were stress reactions to appraisals the stressors and ultimately lead to more depressive symptoms.

This study found that PBJW played a mediating role between emerging adults’ neuroticism and depressive symptoms. The core characteristics of neuroticism included exaggerated negative emotionality, the belief that the world was a dangerous and threatening place, and the perception of inadequate coping toward challenging events (Barlow et al., 2014). The perception that the world was dangerous and threatening place obviously hinders emerging adults’ belief that the world would treat them fairly—that is, their PBJW. According to the just world theory, the tendency to perceive the world as more just for oneself than for others functions as a self-protective mechanism that allows emerging adults to view themselves as relatively privileged persons (Lerner, 1980). As an important personal coping resource, PBJW could influence the way that emerging adults cope with daily events and challenges (Dalbert, 2001), and finally facilitate their mental health. The present study revealed that PBJW may be a possible reason for the negative relation between neuroticism and depressive symptoms among emerging adults.

In addition, the findings confirmed the hypothesis that forgiveness mediated the relation between emerging adults’ neuroticism and depressive symptoms. Studies showed that emerging adults with high neuroticism were likely to produce more intensified negative affect and ruminate on a negative state for a longer time (Dunkley et al., 2014; Riek and Mania, 2012), which may decrease their level of forgiveness. Forgiveness could prompt emerging adults to reframe negative events as neutral or positive, which benefited psychological well-being and prevented depressive symptoms (Tse and Yip, 2009). Therefore, emerging adults with higher neuroticism tended to be lower in forgiveness, and the resulting decrease in forgiveness led to more depressive symptoms.

More importantly, this study further confirmed the sequential mediation effect of PBJW and forgiveness in the relation between emerging adults’ neuroticism and depressive symptoms. This finding was consistent with previous studies reporting a positive relation between PBJW and forgiveness (Bartholomaeus and Strelan, 2016; Strelan and Sutton, 2011). PBJW encouraged recognition of the value of using forgiveness to restore a just world (Bartholomaeus and Strelan, 2016); hence, emerging adults with high PBJW were grateful for the contributions of others and were more likely to be forgiving. Conversely, emerging adults with low PBJW who thought the world was unjust appeared to be less forgiving (Strelan, 2007). Therefore, emerging adults’ neuroticism was not only directly positively related to depressive symptoms but also reduced PBJW, thus reducing the level of forgiveness and ultimately leading to more depressive symptoms.

In sum, the relation between neuroticism and depressive symptoms was undoubted, however, neuroticism was a relatively stable personality trait that was difficult to change to decrease depressive symptoms. Revealing its association with depressive symptoms was mediated by modifiable coping strategies was particularly relevant for prevention and intervention programs. The findings indicated that prevention and intervention strategies in depressive symptoms of emerging adults’ with high neuroticism could focus on engaging in specific adaptive coping strategies such as PBJW and forgiveness. Specifically, PBJW, as an adaptive internal coping resource, was important for dealing with difficulties. Guidance could be provided for emerging adults to gain a correct understanding of challenges and unfairness so that they could develop stronger PBJW. In addition, forgiveness was a way of letting go of past difficulties and their aftermath (Carbonell et al., 2005). The ability to “let go” was recommended as part of emotional healing after negative experiences (Kaminer et al., 2000). A particular focus could be placed on improving emerging adults’ forgiveness. In sum, interventions that foster PBJW and forgiveness will provide emerging adults with sufficient internal resources when they attempted to respond to challenges and adversities. Previous studies paid more attention to the maladaptive coping strategies or the general adaptive coping ability when exploring the mechanism between neuroticism and depressive symptoms. This study provided a new insight by focusing on two specific adaptive coping resources. The findings suggested that neuroticism predisposed emerging adults to have a lower level of adaptive coping ability. Improving adaptive coping ability—especially PBJW and forgiveness—was effective in reducing their depressive symptoms.

Although the findings contributed to our understanding of the onset mechanism of depressive symptoms, there were still limitations of this study. First, it was noteworthy that PBJW was shaped by cultural traditions. In Chinese cultures, karma——a widely held belief——may play an important role in shaping emerging adults’ PBJW. An empirical study found that karma was associated with PBJW, particularly in cultures where karmic beliefs prevail (White et al., 2019). This implied that cultural context may serve as a moderator in the relation between PBJW and other variables. Consequently, the associations between these variables might differ across contexts, depending on the prevalence of karmic beliefs. Given these considerations, future studies could investigate the relations between these variables in culturally diverse samples to examine cultural generality of the findings. Second, this study was limited by the potential confounding effects of additional variables (e.g., social support, moral cognition, or empathy) that may overlap with forgiveness. Future research could control for these variables when examining forgiveness as the target construct. Third, we recruited only young adults from the general population rather than clinical samples. Future studies should replicate this study among clinical samples to extend the generalizability of the results. Forth, the cross-sectional nature of this study prevented us from drawing conclusions about the direction and causality of the results. Therefore, to further test the causal relationship among these four variables, it was necessary to conduct longitudinal studies in the future. Last, we used self-report measures to obtain the data. Although common method variance was not a serious problem in the present study, it may inflate associations. Hence, there was a need for greater use of informant reports and observational measures in future studies.

In conclusion, the separate mediation effects of PBJW and forgiveness and sequential mediation effect of these two variables were significant. This study provided an in-depth understanding of the onset mechanism of emerging adults’ depressive symptoms. Although high neuroticism was a stable predictor of the risk of depressive symptoms, PBJW and forgiveness, as adaptive coping mechanisms, represented an effort to exert control and act to overcome stress (Yin et al., 2017) and may relieve depressive symptoms during emerging adulthood.

## Data Availability

The raw data supporting the conclusions of this article will be made available by the authors, without undue reservation.
